# Behavioral and Resting State Functional Connectivity Effects of High Frequency rTMS on Disorders of Consciousness: A Sham-Controlled Study

**DOI:** 10.3389/fneur.2018.00982

**Published:** 2018-11-21

**Authors:** Xiaoyan Liu, Fanxia Meng, Jian Gao, Li Zhang, Zhen Zhou, Gang Pan, Benyan Luo

**Affiliations:** ^1^Department of Neurology, The First Affiliated Hospital, College of Medicine, Zhejiang University, Hangzhou, China; ^2^Department of Rehabilitation, Hangzhou Hospital of Zhejiang CAPR, Hangzhou, China; ^3^Department of Rehabilitation Medicine, Zhejiang Provincial People's Hospital, Hangzhou, China; ^4^Department of Computer Science and Technology, Zhejiang University, Hangzhou, China

**Keywords:** default mode network (DMN), disorders of consciousness (DOC), functional connectivity, minimally conscious state (MCS), resting state, transcranial magnetic stimulation

## Abstract

**Objectives:** A combined approach of behavioral characteristics and network properties was applied to explore the effect of repetitive transcranial magnetic stimulation (rTMS) on disorders of consciousness (DOC) and to observe changes in brain network connections before and after the stimulation.

**Methods:** A total of 7 DOC patients and 11 healthy controls were enrolled. The study was designed as a randomized, sham-controlled study. All DOC patients were given 20 Hz rTMS real and sham stimuli to the left M1 region, with each stimulus lasting for 5 consecutive working days and the interval between two stimuli being 1 week. Coma Recovery Scale-Revised (CRS-R) and resting state functional MRI data before and after stimuli were collected. The functional connection (FC) of the default mode network and the frontoparietal network were chosen as the central target to compare differences in network connections between the DOC group and the normal control group. For DOC patients, changes in behavior and brain function before and after real and sham stimuli were also assessed as a group and individually.

**Results:** (1). The overall analyses showed no significant changes of CRS-R scores or brain FC following real or sham rTMS stimuli in the DOC patients. However, real rTMS stimuli tended to enhance the FC of nodes in left lateral parietal cortex (LPC), left inferior temporal cortex (ITC) and right dorsolateral prefrontal cortex (DLPFC). (2). The individual analyses showed one minimally conscious state (MCS) patient presented with a obviously increased CRS-R score following real rTMS stimuli, and a visibly enhanced connectivity was observed in the nodes of left LPC, left ITC and right DLPFC of this patient.

**Conclusion:** Our findings did not provide sufficient evidence of therapeutic effect of 20 Hz rTMS over the left M1 in DOC. However, MCS patients shortly after brain injury may possibly benefit from rTMS. Reconstruction of the left LPC, the left ITC and the right DLPFC may be the brain networking foundation of improvements in consciousness from rTMS.

## Introduction

The term chronic disorders of consciousness (DOC) refers to the state of wakefulness without awareness or with minimal awareness in a patient following brain injury, including the unresponsive wakefulness syndrome/vegetative state (UWS/VS) and the minimally conscious state (MCS) (1). To be noted, much effort has been made to explore the effects of drugs and neuromodulation on DOC in recent years (2–5), however, very few affirmative results or means of evaluation have been achieved.

Transcranial magnetic stimulation (TMS) is a non-invasive, easy to operate neural stimulating technique, posses the property of detecting and modulating neurological and psychological disorders. Given its property of detection, Rosanova et al. has applied a combination of TMS and high-density electroencephalograph (EEG), to detect effective connectivity of non-communicating subjects and thus reliably measure their consciousness level (6). Given its property of modulation, high frequency repetitive TMS (rTMS) increases the excitability of cortical neurons, whereas low frequency rTMS decreases their excitability. These effects persist for a period following the stimulation (7). High frequency rTMS has lately been used in an attempt to treat DOC, with the stimulation points mostly positioned at the primary motor cortex (M1) or the dorsolateral prefrontal cortex (DLPFC). Xia et al. discovered that 10 Hz rTMS applied to the left DLPFC could decrease low frequency power and increase high frequency power in DOC patients, particularly in MCS patients (8). Manganotti et al. applied 20 Hz rTMS to the M1 region in 3 UWS/VS patients and 3 MCS patients and achieved significant behavioral improvement in one of the MCS patients, whose Coma Recovery Scale-Revised (CRS-R) score increased by 8 points and whose EEG also persistently responded well (9). However, in another randomized and sham-controlled EEG study conducted by Cincotta et al., there was no improvement of consciousness in UWS/VS patients following 20 Hz rTMS applied at the M1 region (10).

Although CRS-R has been used for many years as a commonly accepted means to evaluate consciousness and behavior, it incorporates a certain degree of subjectivity. Better yet, there is a rapidly growing literature highlighting neuroimaging technology as an objective tool for evaluating DOC. Stender et al. examined the resting metabolic activity of DOC, and proposed cerebral ^18^F-FDG PET had an emerging possibility to identify MCS patients and predict recovery of UWS/VS patients (11). Meanwhile, the technique of the resting state functional MRI (fMRI) has broadened neuroimaging indicators. Through this method, the severity of DOC can be identified in the internal and external connections of the default mode network (DMN) (12), decreased connectivity has been frequently described in the DMN among DOC patients and has been suggested to be in proportion to the degree of DOC (13–16). Moreover, changes in brain network connections before and after stimuli may also be observed dynamically (17). In this study, we intended to combine behavioral characteristics and brain network properties shown by resting state fMRI to investigate the interventional effect of rTMS on DOC.

## Methods

### Subjects

Seven patients who had suffered from a severe closed head injury were enrolled in this study. They had emerged from coma. The control group consisted of 11 healthy volunteers. All patients were recruited from the Department of Rehabilitation in the Hangzhou Hospital of Zhejiang, Hangzhou, China, fulfilled the international standard established for MCS or UWS/VS (18, 19), and met the following inclusion criteria: (1) no use of drugs modifying cortical-excitability (beyond L-DOPA, baclofen and anti-epileptic drugs); (2) no use of sedatives or neuromuscular blockers within 24 h; (3) no history of epilepsy, other severe neurological or systemic disease; (4) no critical condition (respiratory or hemodynamic instability); (5) no contraindication to TMS; (6) open eyes; (7) right handed.

Each patient was evaluated using the CRS-R score which was used to assess the level of consciousness, covering the following 6 functions: auditory, visual, motor, verbal, communication and arousal function, with the total scores ranging from 0 to 23 points. The demographic data (6 male patients, median age 48 ± 16.57 years) and clinical characteristics of these 7 patients are shown in Table 1. For the 11 normal controls (6 male, age 46 ± 18.5 years), in Table 2, there was no history of mental, neurological or systemic disease, no history of drug or alcohol abuse, no history of brain injury. This study followed the Helsinki Declaration and was approved by the Ethics Committee of the First Affiliated Hospital of Zhejiang University School of Medicine. An Informed Consent Form was signed by the legal representative of each patient prior to the study.

**Table 1:** Demographic and clinical characteristics of the 7 brain-injured patients.

**Patient**	**Age range (years)**	**Etiology**	**Months after injury**	**Clinical assessment**
P1	60–65	Trauma	6	MCS
P2	10–15	Anoxia	4	MCS
P3	46–50	Trauma	5	UWS/VS
P4	56–60	Hemorrhage	2	UWS/VS
P5	40–45	Trauma	1	MCS
P6	60–65	Trauma	2	MCS
P7	50–55	Trauma	2	MCS

**Table 2:** Demographic characteristics of the 11 healthy controls.

**Healthy control**	**Age range (years)**	**Gender**
C1	50–55	Female
C2	60–65	Female
C3	26–30	Male
C4	16–20	Female
C5	20–25	Male
C6	56–60	Male
C7	66–70	Female
C8	26–30	Male
C9	50–55	Male
C10	60–65	Female
C11	56–60	Male

### Protocol of the study and mode of stimulation

This study was designed as a randomized, sham-controlled study. Real or sham 20 Hz rTMS stimuli were randomly applied to the DOC patients one after another. Each period of stimulation lasted for 5 consecutive working days (Monday to Friday 8:00 to 10:00). The interval between two stimulation periods was one week. Using a Magstim-Rapid2 stimulator (Magstim Company Ltd., London, UK), rTMS was delivered in a posteroanterior direction over the left M1 through a figure-of-eight focal coil oriented on the basis of resting motor threshold (RMT), which was defined in accordance with the International Federation of Clinical neurophysiology Committee recommendations in advance (20), namely, determined as the minimum stimulus intensity that induced a stretch of the contralateral thumb in at least five of 10 consecutive trials during muscle relaxation. If the RMT was above 67%, then the actual intensity of the stimulus was set to 60% of the maximum output of the stimulation device (21). For rTMS real stimuli, 1000 pulses were delivered in 20 arrays at a frequency of 20 Hz, with each array lasting 2.5 s and a pause of 28 s in between. The intensity of the stimuli equaled to RMT. For rTMS sham stimuli, the parameters were the same as above, but were 90° angled away from patients' head.

A double-blind approach was used. All DOC patients receiving real or sham stimuli were evaluated for their CRS-R scores by a clinician at the following time points: baseline time before first stimulation cycle (T0), immediately after the first stimulation cycle (T1), and 48 h after first stimulation cycle (T2). After a 1-week interval there was a second stimulation cycle. A baseline time was before the second stimulation cycle (T0), immediately after second stimulation cycle (T1), and 48 hours after second stimulation cycle (T2). In addition, resting state fMRI data were acquired at the following time points: baseline time before first stimulation cycle (T0), Immediately after first stimulation cycle (T1) and immediately after second stimulation cycle (T1). Baseline resting state fMRI data of the healthy controls were also acquired.

### MRI data acquisition

MRI scans were performed on a Siemens 1.5T MRI scanner. The subject lay flat on the examination bed, with a head frame (a routine part of the MRI scanner) placed at the temporal position to restrict head movement. High resolution whole brain T1-weighted images were acquired through T1-weighted 3D magnetization prepared rapid acquisition gradient echo sequences: Repetition Time 2,000 ms, Echo Time 5.18 ms, Flip Angle 15°, Matrix 256 × 256, Field of View 240 × 240 mm, Thickness 1.0 mm, and Gap 0.5 mm. Whole brain T2-weighted functional imaging data were acquired through gradient-echo echo-planar imaging sequences and axial scanning with AC-PC as the plane of reference: Repetition Time 2000 ms, Echo Time 40 ms, Flip Angle 90, Matrix 64 × 64, Field of View 240 × 240 mm, Thickness 5.0 mm, and Gap 1.0 mm. In total 240 scanning time points were obtained during MRI scan which lasted for 8 min.

### Resting state fMRI data pre-processing

Resting state fMRI images were pre-processed with the Data Processing & Analysis for Brain Imaging toolkit (DPABI 3.1) (http://rfmri.org/dpabi) (22). The first ten volumes of the functional phase were removed to ensure T1 equilibrium and adaptation of the subject toward the scanning environment. The remaining images were subjected to time correction and motion correction. Any motion >2.5 mm in any direction or rotation >2.5 degree were regarded as excessive head movement, and P3 following real rTMS stimuli as well as P6 and P7 following sham rTMS stimuli were excluded. To further control the head motion effect, two-sample *t*-test was used to compare the group differences in head motion using the means of frame-wise displacement, but no significant differences were demonstrated between DOC patients and normal controls (*t* = 0.412, *P* = 0.686), or between sessions of DOC patients (real T1 vs.T0: *t* = −0.171, *P* = 0.871; sham T1 vs. T0: *t* = 0.633, *P* = 0.561). Then nuisance covariates were regressed out from the motion corrected fMRI data, including average white matter signals and cerebrospinal fluid signals, as well as 6 rigid motion parameters. The remaining images underwent spatial normalization with re-sampled to 3 × 3 × 3 mm voxels, Gaussian kernel function was performed with full width at half maximum (FWHM) set to 6 mm for image smoothing, and detrending was then conducted to remove the linear trend of time courses. Finally, the resting state fMRI data analysis toolkit (RESTplus 1.2) (http://www.resting-fmri/) was used for band-pass filtering, so that the blood oxygen level-dependent time sequences were maintained at low frequency oscillation of 0.01–0.08 Hz.

### Functional connectivity (FC) images

**Figure 1:**
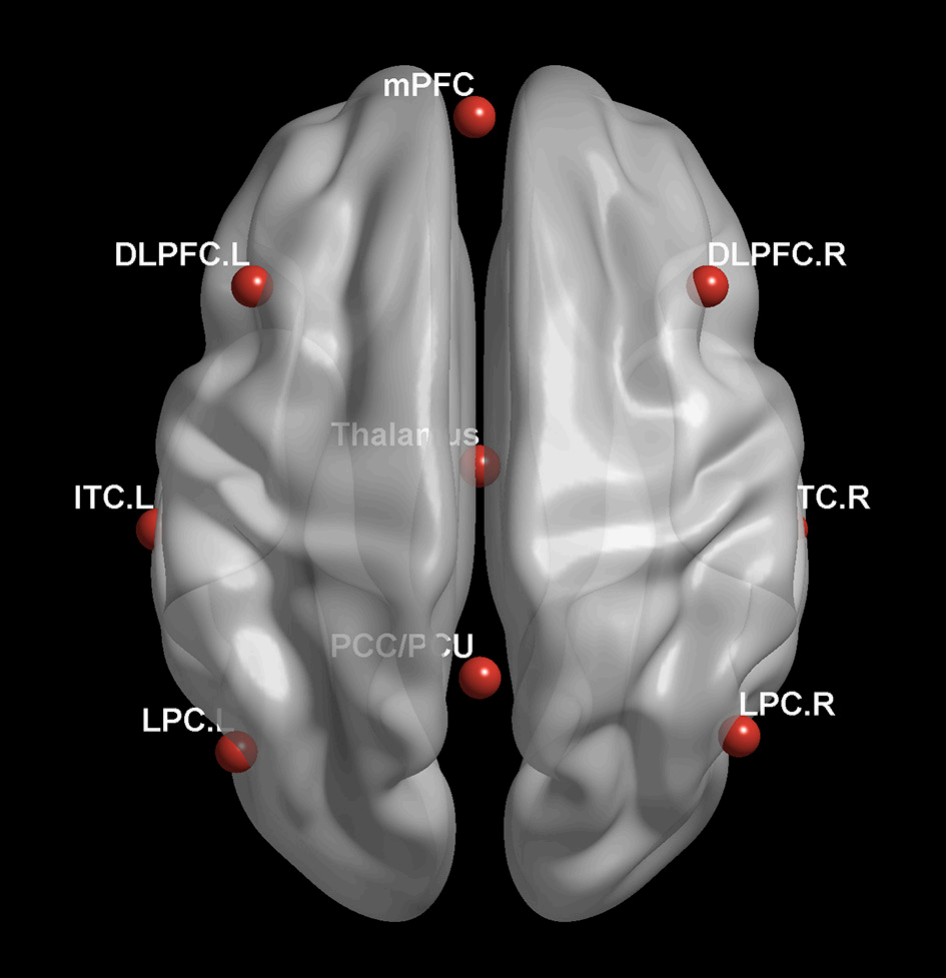
Selected Regions of interest within the DMN and the frontoparietal network.

The RESTplus 1.2 software package was used to obtain FC data. With a reference to previous literature and with careful consideration of the correlation between internal or external network and consciousness level or clinical outcome, the authors chose the DMN and the frontoparietal network as the regions of interest (ROI), which included the posterior cingulate cortex/precuneus (PCC/PCU) [0–52.27], the medial prefrontal cortex (mPFC) [-1 54 27], the left lateral parietal cortex (LPC) [−46 – 66 30], the right LPC [49–63.33], the left inferior temporal cortex (ITC) [-61−24−9], the right ITC [58−24−9], the thalamus [0−12 9], the left dorsolateral prefrontal cortex (DLPFC) [-43 22 34] and the right DLPFC [43 22 34], in Figure 1. Based on voxel-wise FC analyses, the correlation between the average time sequence and the whole brain voxel time sequence was calculated for each ROI. The FC graphs obtained thus were then converted to Z-maps with the Fisher's r-to-z transformation, and the whole brain Z-maps for each ROI was then plotted for each subject.

### Statistical analyses

As the first step, the SPSS13.0 software package was used for analyses of clinical data. The independent sample *t*-test or non-parametrical Mann-Whitney U test was used to analyze the differences in quantitative indicators, for example age, between the DOC patients and the healthy controls. On the other hand, Fisher's exact test was performed to examine difference in gender between the two groups. To eliminate any carryover effect, namely, effect induced by rTMS in the first cycle remains and is brought forward to the second cycle, the Wilcoxon signed ranks test was performed to compare the CRS-R total scores before the first cycle and the second cycle. To eliminate any treatment sequence influence, the Mann-Whitney U test was performed to compare the difference in the results of CRS-R total scores at T2 minus CRS-R total scores at T1 between the group receiving real stimulation followed by sham stimulation and the group receiving sham stimulation followed by real stimulation. Following eliminations of carryover effect and treatment sequence influence, the Wilcoxon signed ranks test was again performed to examine the influence of rTMS on the CRS-R total score (rTMS real stimuli: T0 vs. T1, T0 vs. T2; rTMS sham stimuli: T0 vs. T1, T0 vs. T2). A value of *p* < 0.05 was regarded as statistically significant.

The RESTplus 1.2 software package was also used for analyses of the FC data. Firstly, differences in degree of whole brain connectivity with each ROI between the DOC patients and the control group were examined with independent sample *t*-test. Secondly, in order to identify any cerebral functional changes that might be caused by rTMS amongst the DOC patients, paired *t*-test was also performed to examine FC differences between each ROI with the whole brain of real rTMS at T0 and T1, and of sham rTMS at T0 and T1. The data were further calibrated with a Gaussian Random Field Theory Multiple Comparison Correction (voxel level *p* < 0.001, cluster-level *p* < 0.05, two tailed, with masking).

## Results

### Clinical characteristics of the subjects

The clinical characteristics of the 7 DOC patients (5 MCS and 2 UWS/VS) are shown in Table 1. There are no statistical differences in age (*t* = 0.281, *p* = 0.782) or gender (*p* = 0.316) between these patients and the 11 healthy controls. The individual CRS-R total score at each time point and the score of each item are shown in Table 3. No significant side effects from rTMS were observed in this study.

**Table 3:** Individual data of the CRS-R total and subscales scores at each time point.

	**Real rTMS**			**Sham rTMS**		
	**T0**	**T1**	**T2**	**T0**	**T1**	**T2**
P1	6	7	7	7	7	7
	(1,2,1,0,0,2)	(1,2,1,0,0,3)	(1,2,1,0,0,3)	(1,2,1,0,0,3)	(1,2,1,0,0,3)	(1,2,1,0,0,3)
P2	16	16	16	16	16	16
	(4,5,2,1,1,3)	(4,5,2,1,1,3)	(4,5,2,1,1,3)	(4,5,2,1,1,3)	(4,5,2,1,1,3)	(4,5,2,1,1,3)
P3	7	7	7	7	7	7
	(1,1,2,1,0,2)	(1,1,2,1,0,2)	(1,1,2,1,0,2)	(1,1,2,1,0,2)	(1,1,2,1,0,2)	(1,1,2,1,0,2)
P4	6	7	7	6	6	6
	(1,1,1,1,0,2)	(1,1,2,1,0,2)	(1,1,2,1,0,2)	(1,1,1,1,0,2)	(1,1,1,1,0,2)	(1,1,1,1,0,2)
P5	15	23	23	15	15	15
	(2,4,4,2,1,2)	(4,5,6,3,2,3)	(4,5,6,3,2,3)	(2,4,4,2,1,2)	(2,4,4,2,1,2)	(2,4,4,2,1,2)
P6	6	7	7	7	7	7
	(1,1,2,0,0,2)	(1,1,2,1,0,2)	(1,1,2,1,0,2)	(1,1,2,1,0,2)	(1,1,2,1,0,2)	(1,1,2,1,0,2)
P7	13	13	13	13	13	13
	(2,4,4,1,0,2)	(2,4,4,1,0,2)	(2,4,4,1,0,2)	(2,4,4,1,0,2)	(2,4,4,1,0,2)	(2,4,4,1,0,2)

### Comparison between DOC patients and healthy controls

Our data show that, by comparison with the healthy controls, the DOC patients demonstrated significantly altered FC. In particular, those DOC patients exhibited enhanced connections between the node of right LPC and the left precentral gyrus/postcentral gyrus, in contrast, they had weakened connections between the node of left ITC and the left cuneus/superior occipital gyrus, between the node of thalamus and the right medial frontal gyrus/anterior cingulate gyrus, also between the node of left DLPFC and the left cerebellum posterior lobe (all *p-*values after correction < 0.001). In addition, a trend toward altered FC was indicated among DOC patients in the nodes of PCC/PCU, mPFC, left LPC, right ITC, and right DLPFC, no significant differences yet achieved (those *p-*values after correction >0.001).

To sum up, compared to healthy controls, DOC patients presented with significantly altered connectivity in several brain regions of the DMN and the frontoparietal network, including the right LPC, left ITC, thalamus, and left DLPFC.

### Regulatory effect of 20 hz rTMS on DOC: measured by CRS-R scores

At the group level, there was no significant difference in the CRS-R total scores before real rTMS or sham rTMS (*Z* = −1.414, *p* = 0.157), thus eliminating the carryover effect. To supplement this, an individual evaluation was performed, actually, real rTMS, not sham rTMS, was applied to P1, P3, P6, and P7 at the first step, resulting in the CRS-R total score elevated by 1 in P1 but not increased in other three patients. A comparison between the group receiving real stimulation followed by sham stimulation and the group receiving sham simulations followed by real simulation revealed no difference in post-simulation CRS-R total scores (*p* = 0.445), thus eliminating the treatment sequence influence also.

Although the results of the overall behavioral study indicated a slight improvement in CRS-R total scores of the DOC patients following real rTMS, the difference was insignificant (*Z* = −1.890, *p* = 0.059). On the other hand, there was no significant improvement in CRS-R total score following sham simulation (*Z* = 0.000, *p* = 1.000). The results of the individual behavioral study indicated that the CRS-R total score was improved in one MCS patient (P5) whose DOC had lasted for 1 month. In this case, the scores for auditory, visual, motor, verbal, communication and arousal functions were all improved, from 2, 4, 4, 2, 1, and 2 points to 4, 5, 6, 3, 2, and 3 points, respectively. In addition, the CRS-R total score increased from 15 to 23. For the same patient, following sham stimulation, there was no improvement in either CRS-R total score or individual score for each of the above items.

### Regulatory effect of 20 hz rTMS on DOC: measured by FC data

To investigate possible regulatory effect of rTMS on DOC and explore the mechanism thereof, a multitude of ROIs in the DMN and the frontoparietal network were chosen as the targets, including the PCC/PCU, the mPFC and the thalamus, as well as the LPC, ITC, and DLPFC of both sides.

The results of an overall longitudinal study show that, compared to the findings before stimulation, FC was not significantly enhanced in the DOC patients following real rTMS (all *p-*values after correction > 0.001). Nevertheless, a trend toward enhanced FC was indicated, including that the node of left LPC with the right cingulate gyrus and inferior frontal cortex; the node of left ITC with the cuneus; and the node of right DLPFC with the right cerebellum. In contrast, after sham rTMS, when compared with that before stimulation, neither significant enhancement of brain network in the ROIs, nor a enhanced trend was revealed in the DOC patients.

**Figure 2:**
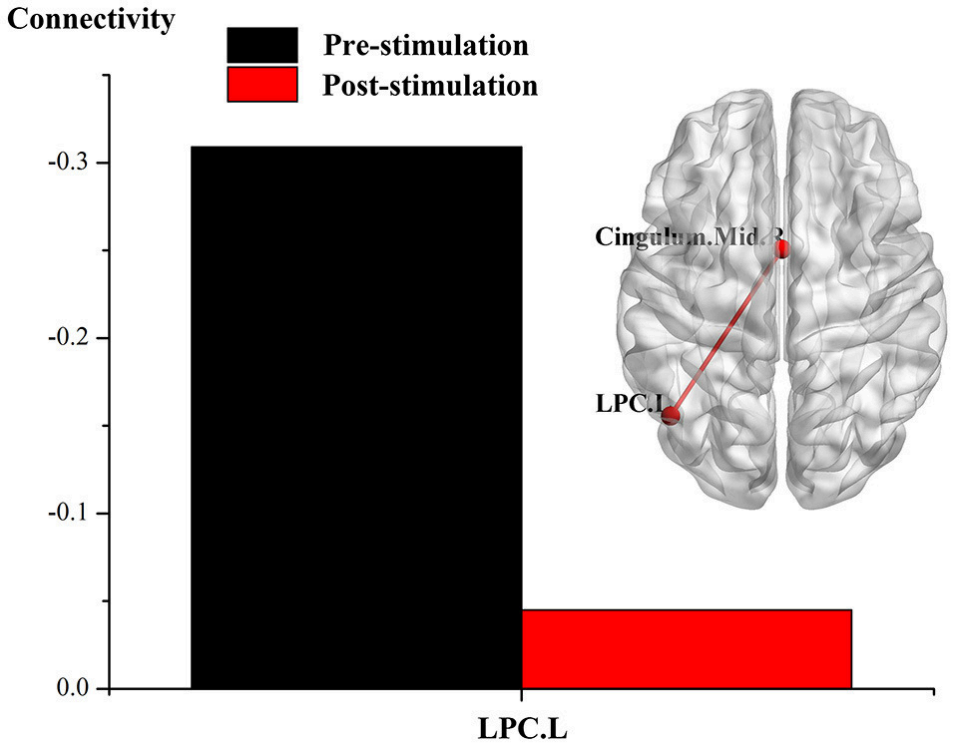
Effect of real rTMS on the degree of whole brain connectivity of the DMN and the frontoparietal network of patient 5 (P5). (1) ROI is left LPC; (2) ROI is left ITC; (3) ROI is right DLPFC.

**Figure 3:**
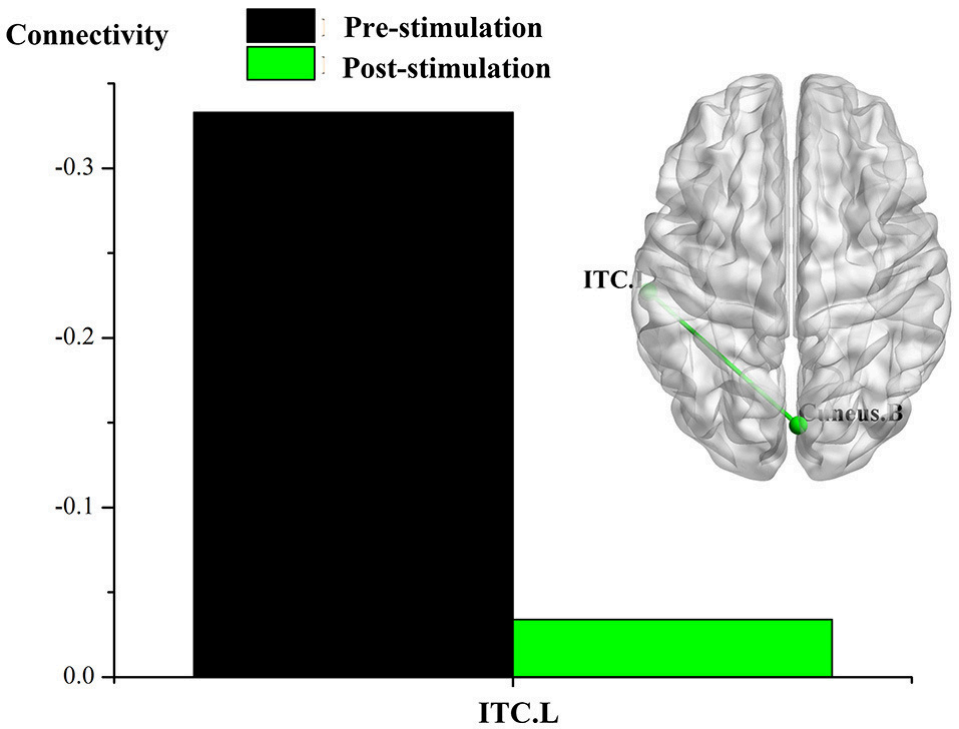
Effect of real rTMS on the degree of whole brain connectivity of the DMN and the frontoparietal network of patient 5 (P5). (1): ROI is left LPC; (2): ROI is left ITC; (3): ROI is right DLPFC.

**Figure 4:**
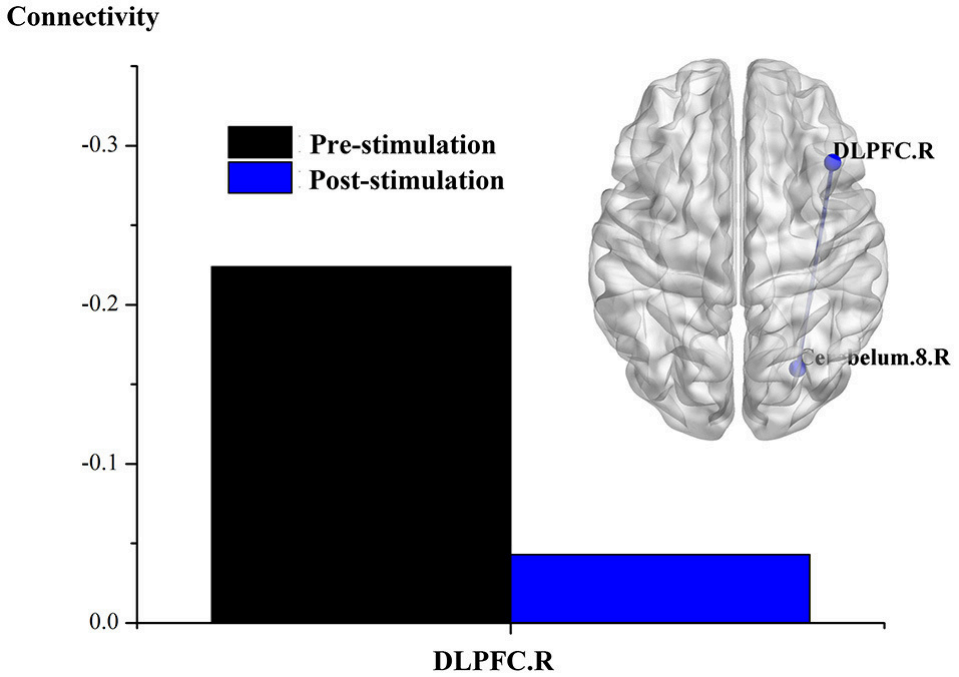
Effect of real rTMS on the degree of whole brain connectivity of the DMN and the frontoparietal network of patient 5 (P5). (1): ROI is left LPC; (2): ROI is left ITC; (3): ROI is right DLPFC.

The results of individual longitudinal study also confirm, that for the MCS patient (P5) with significant improvement of consciousness in terms of behavioral study, FC value was increased for the connectivity of the left LPC with the right cingulate gyrus and the inferior frontal cortex, the left ITC with the cuneus, and the right DLPFC with the right cerebellum, see Figures 2–4.

## Discussion

In this study, for the first time, a behavioral study is combined with resting state fMRI scanning to examine changes in brain network functions induced by rTMS in DOC patients. The results show that following real rTMS, one patient (P5) who had been in MCS for 1 month was improved in auditory, visual, motor, verbal, and communication and arousal functions, especially auditory and motor functions. Along with developments in neuroimaging technology, resting state fMRI has become a powerful method for exploring potential mechanisms and identifying biomarkers for neurological diseases (23), it has also gradually become an objective indicator for evaluating DOC. Using FC technique of resting state fMRI, we demonstrated 3 main findings: (1) Compared to the healthy controls, DOC patients presented with several brain regions of significantly altered FC in the DMN and the frontoparietal network. (2) Overall, real rTMS had no significant influence on FC value of the selected ROIs, but aroused a trend toward enhanced FC in the nodes of left LPC, left ITC and right DLPFC. (3) Individually, the changes in brain network connectivity presented by the MCS patient showing behavioral improvement were consistent with the findings from the overall study.

According to the Information Theory, consciousness equals to the capacity of the brain to integrate information (24). From the viewpoint of resting state fMRI, the capacity of the brain to integrate information depends entirely on the degree of connectivity of those brain networks associated with the generation and maintenance of consciousness. Previous studies have indicated that the level of consciousness and recovery from DOC are both related to functional connections of the DMN, and that the DMN may be regarded as a possible marker in the explanation of the recovery of consciousness (25). Furthermore, studies also indicate that consciousness as a whole consists of internal and external consciousness; and internal consciousness is associated with the DMN (26), whereas external consciousness is associated with the frontoparietal cortex network (27). To refine the study of this subject and to explore the brain network mechanism of the effect of short-term rTMS on consciousness, we opted to select multiple nodes in the DMN and the frontoparietal cortex network to observe changes of brain network connectivity before and after rTMS. The targets included the PCC/PCU, the mPFC, the thalamus, and the bilateral LPC, ITC, and DLPFC.

It is revealed in this study, that patients in DOC showed enhanced connections in the node of right LPC, as well as weakened connections in the nodes of left ITC, thalamus and left DLPFC. It is commonly accepted that the LPC is associated with the functions of attention, multiple mode integration, subjective extraction and action-related consciousness (28), and that the ITC corresponds to information reception and is directly associated with visual recognition and visual-perception-related consciousness (29). The altered connectivity of the above two networks is not only the actual representation of altered internal consciousness, but also the brain network mechanism for the enhancement of action-related and visual-perception-related consciousness. Because of the role of DLPFC in executive function and complex mental activities, it is not difficult to understand the rationale behind the selection of DLPFC as the typical ROI of the frontoparietal network in this study. Since DOC patients presented weakened FC between DLPFC and cerebellum, we assume that the effect of cerebellum on consciousness modulation will depend on the degree of its connectivity to the frontoparietal network. This assumption is consistent with cerebellum functional reconstruction in the recovery of consciousness (30). Further studies are needed to explore the exact function of cerebellum in the field of DOC. On the other hand, data emerged from previous studies indicates a fundamental brain mechanism underlying consciousness is related to two primary patterns of brain activity, the medial parietal cortex/posterior medial complex and the thalamus, which are likely to be the reflections of neuronal activity within the corticothalamic systems (31, 32). In line with it, altered nodes between DOC patients and healthy controls in the current study were all within the corticothalamic systems.

It is worthy mentioning, that no significant FC alteration in the nodes of highlighted PCC/PCU or mPFC was discovered in our DOC patients, 5 in MCS and 2 in UWS/VS, in a comparison with healthy controls. Our previous data have showed that various degrees of DOC possess various FC patterns, in this regard, DOC patients own reduced PCC/PCU connectivity and increased mPFC connectivity, however, MCS patients show increased mPFC connectivity but without decreased PCC/PCU connectivity, while UWS/VS patients reveal weakened PCC/PCU connectivity but without increased mPFC connectivity (33). Moreover, it has been indicated that the DMN connectivity is weakened in severely brain-damaged patients, in relation to the degree of consciousness impairment (13). Taken together, these results support a notion that the consciousness-related processes of disruption and remodeling are possibly imbalance throughout the neuronal system and related to consciousness level.

In this study, real 20 Hz rTMS stimuli over the left M1 lasted for 5 consecutive working days were well-tolerated. Nevertheless, when the whole group of DOC patients was considered, it did not produce neither significant behavioral improvements, as detected by CRS-R, nor significant brain FC changes, as defined within the nodes of PCC/PCU, mPFC, LPC, ITC, thalamus and DLPFC. Two hypotheses could be proposed to illustrate the inefficacy of the current rTMS stimuli to DOC patients. First, a particular limitation in the field of DOC study is the heterogeneity of DOC patients that has an influence on clinical efficacy of rTMS treatment, including various etiologies, various levels of consciousness impairment, various time to evaluation. Those of the current study were varied, etiologies including trauma, anoxia and hemorrhage, time after injury ranging from 1 to 6, and severity involving MCS and UWS/VS. Second, 5 consecutive working days may not be sufficient, and the left M1 may not be the ideal target. However, as it indicated in several studies, rTMS or tDCS applied to the DLPFC could not always produce consciousness improvement among the DOC patients (34, 35). Namely, few data could support a better target over the M1 at present.

Nevertheless, a trend toward enhanced FC was detected following real rTMS, including the nodes of left LPC, left ITC and right DLPFC. Furthermore, we focused on longitudinal behavioral changes and FC alterations after rTMS stimuli at the individual level. As a result, P5 in a state of MCS was clinically improved. From the perspective of what kind of DOC patients benefit more from rTMS stimuli, we explored the characteristics of P5, indicating an etiology of trauma, with shortest interval time and in a state of MCS. We further explored the brain responses accompanied by clinical improvement following rTMS stimuli. The brain responses were measured by FC alteration of the defined nodes. As a consequence, enhanced FC was defined in the nodes unified with overall study, including the nodes of left LPC, left ITC, and right DLPFC. Those data indicated, that a combination of overall and individual data, integrating longitudinal behavioural and brain function evaluation, could properly evaluate the rTMS efficacy and its underlying neural mechanism. In accordance with it, a previous study has performed a longitudinal single case behavioural and task fMRI of the DOC, and suggested to conduct a longitudinal evaluation in determining the clinical efficacy (36). Considering the underlying mechanisms, previous studies have indicated that, in a comparison with healthy controls, brain regions of reduced connectivity in DOC patients included the LPC (25, 33), and that follow-up of UWS/VS patients without neuromodulation treatment confirmed a lower degree of FC in LPC in patients without clinical improvement than in those with improvement (37, 38). We thus suggest that the potential neural network mechanism of rTMS treatment may be the same as the mechanism for spontaneous recovery of DOC patients.

However, there are some limitations in this study. Firstly, the sample size is relatively small, which may lead to a low reliability of the treatment effect of rTMS. To overcome this, longitudinal clinical and resting state fMRI data were also collected, complex analytical methods were used to control bias, overall and individual level analysis were performed. Secondly, the etiology of the DOC for the subjects in this study was not unified. Thus, stratified analyses may be required in further study. Thirdly, only a regimen of short-term stimulation with 1-week interval was used in this study. Thus, flaws may still exist despite elimination of carryover effect. As long-term stimulation may produce a better treatment effect and the effect of rTMS may last over 1 week, a regimen with a prolonged interval may be better in future studies. Meanwhile, both short-term and long-term effects should be evaluated.

All in all, although the current findings could not provide sufficient evidence of therapeutic effect of 20 Hz rTMS over the left M1 in DOC, the results of this study indicate, MCS patients shortly after brain injury may possibly benefit from rTMS, reconstruction of the resting state network may be the foundation of consciousness improvements.

## Author contributions

XL, GP, and BL contributed to study design. XL, FM, JG, and LZ contributed to data collection. XL, FM, and ZZ contributed to data analysis and interpretation. XL contributed to manuscript drafting. GP and BL contributed to manuscript revising and responsibility for conduct of research and final approval.

### Conflict of interest statement

The authors declare that the research was conducted in the absence of any commercial or financial relationships that could be construed as a potential conflict of interest.
